# Gross genomic alterations and gene expression profiles of high- grade serous carcinoma of the ovary with and without BRCA1 inactivation

**DOI:** 10.1186/1471-2407-10-493

**Published:** 2010-09-15

**Authors:** Manohar Pradhan, Björn Å Risberg, Claes G Tropé, Matt van de Rijn, C Blake Gilks, Cheng-Han Lee

**Affiliations:** 1 Division of Pathology, Oslo University Hospital, Oslo, Norway; 2 Institute for Medical Informatics, Oslo University Hospital, Oslo, Norway; 3 Department of Gynecologic Oncology, Oslo University Hospital, Oslo, Norway; 4 Faculty Division the Norwegian Radium Hospital, University of Oslo, Oslo, Norway; 5 Department of Pathology, Stanford University Medical Center, Stanford, CA, USA; 6 Department of Pathology and Laboratory Medicine, University of British Columbia and Vancouver General Hospital, Vancouver, British Columbia, Canada

## Abstract

**Background:**

BRCA1 gene inactivation causes chromosomal instability, leading to rapid accumulation of chromosomal rearrangements and mutations. The loss of BRCA1 function due to either germline/somatic mutation or epigenetic silencing is observed in most high-grade serous carcinomas of the ovary.

**Methods:**

DNA ploidy and gene expression profile were used in order to compare gross genomic alteration and gene expression pattern between cases with BRCA1 loss through mutation, BRCA1 epigenetic loss, and no BRCA1 loss in cases of high-grade serous carcinoma with known BRCA1 and BRCA 2 status.

**Results:**

Using image cytometry and oligonucleotide microarrays, we analyzed DNA ploidy, S-phase fraction and gene expression profile of 28 consecutive cases of ovarian high-grade serous adenocarcinomas, which included 8 tumor samples with BRCA1 somatic or germline mutation, 9 samples with promoter hypermethylation of BRCA1, and 11 samples with no BRCA1 loss. None had BRCA2 mutations. The prevalence of aneuploidy and tetraploidy was not statistically different in the three groups with different BRCA1 status. The gene expression profiles were also very similar between the groups, with only two genes showing significant differential expression when comparison was made between the group with BRCA1 mutation and the group with no demonstrable BRCA1 loss. There were no genes showing significant differences in expression when the group with BRCA1 loss through epigenetic silencing was compared to either of the other two groups.

**Conclusions:**

In this series of 28 high-grade serous carcinomas, gross genomic alteration characterized by aneuploidy did not correlate with BRCA1 status. In addition, the gene expression profiles of the tumors showed negligible differences between the three defined groups based on BRCA1 status. This suggests that all ovarian high-grade serous carcinomas arise through oncogenic mechanisms that result in chromosomal instability, irrespective of BRCA status; the molecular abnormalities underlying this in the BRCA intact tumors remains unknown.

## Background

In the western world, ovarian cancer is the leading cause of death among patients with gynecological cancers [1]. High-grade serous carcinoma accounts for 70% of all ovarian cancers, and a disproportionate number of deaths as these tumors are more likely to present with advanced stage disease [2]. Germ line mutations of BRCA1 or BRCA2 genes predispose primarily to high-grade serous carcinoma of the ovary and approximately 16% of high-grade serous carcinoma is associated with germ line BRCA gene mutation [3]. BRCA1 gene inactivation is caused either through mutation or epigenetic silencing by promoter hypermethylation, in contrast to BRCA 2 gene where promoter hypermethylation does not significantly contribute to loss of function [4].

Operating as tumor suppressor genes, the primary function of BRCA genes is to preserve the structural and numerical stability of chromosomes during cell division [5]. The proteins are expressed in the dividing cells and located in the nucleus. BRCA1, by forming a multi-protein complex [6], senses double strand DNA breaks and recruits molecules that repair the breaks by error-free homologous recombination [7,8]. BRCA2, on the other hand, functions as a specific mediator of the interactions leading to homologous recombination [9]. In absence of functional BRCA1 or BRCA2, double stand DNA breaks are repaired by error-prone non-homologous end joining mechanism leading to further mutations and genomic instability [10]. According to the chromosomal instability model for the pathogenesis of BRCA-associated cancers, genetic alterations causing loss of cell-cycle checkpoints and chromosomal instability are crucial during oncogenesis [11,12]. Chromosomal instability can be assessed by degrees of aneuploidy [13] and DNA ploidy related parameters [14]. Gross genomic alteration evidenced by aneuploidy is usually the result of chromosomal instability [15].

Earlier, by analyzing BRCA1 mutation, expression and promoter hypermethylation, we proposed a potential subclassification of high-grade serous adenocarcinomas into three groups: BRCA 1 loss through mutation, BRCA1 epigenetic loss and no BRCA loss [16]. Therapeutically, the subclassification might be useful for tumors susceptible to targeted treatment with inhibitors of poly (ADP-ribose) polymerase (PARP1) [17]. In order to determine associations between BRCA1 loss and gross genomic alteration, tumor proliferation rate and gene expression profile, we have evaluated DNA ploidy and S-phase fraction by high-resolution image cytometry and gene expression profile using oligonucleotide microarrays, in a cohort of high-grade serous carcinomas with defined BRCA1 and BRCA2 status.

## Methods

### Tumors and patients

Samples from the patients with ovarian carcinoma from January 2004 to September 2005 were collected at the Vancouver General Hospital in Vancouver, Canada. The diagnosis of high-grade serous carcinoma was made morphologically and these cases are a subset of those previously reported [16]. Ethical approval was obtained from the University of British Columbia Ethics Board (#H02-61375 and #H03-70606).

### DNA ploidy analysis by image cytometry

Image cytometric DNA ploidy analysis was performed in the cohort as described previously [18]. Briefly, using 50 micron sections of paraffin embedded tissue, a monolayer was prepared and stained with Feulgen method. The images of the nuclei were captured and integrated optical density of individual nuclei was measured using the Fairfield DNA ploidy system. Histograms, made from the integrated optical density, were classified using established criteria [18]. The S-phase fraction was manually calculated by multiplying the number of channels between mid-G0/G1 and mid-G2/M peaks (C) by the mean number of registrations per channel in an even part of the S-phase region (M) and the product was subsequently was divided by the total number of nuclei between the beginning of G0/G1 and the end of G2/M peak (N) expressed in percentage (CxMx100/N) [19]. In the tumors with aneuploid peaks, the S-phase fraction of aneuploid subpopulation was estimated. The S-phase fraction was divided into high and low by using the median as a cutoff point. The DNA index and coefficient of variation (CV) of the peaks were also registered.

### Oligonucleotide microarrays for gene expression profile

The Human Exonic Evidence Based Oligonucleotide microarrays (HEEBO, Stanford) were used to study the global gene expression profiles. Frozen tumors were available for all except two cases of serous carcinomas with no demonstrable BRCA1 loss (case number 208 and 273). Prior to RNA extraction, frozen section analysis was performed and all tumor samples were confirmed to contain viable and representative tumor with no contaminating normal tissue structures. Specimens were subsequently homogenized in Trizol reagent (Invitrogen, Carlsbad, CA, USA) and total RNA was extracted and reverse transcribed into cDNA using a mixture of oligo dT (Operon, HPLC purified) and random hexamer (Amersham, Cat 27-2166-01) primers with incorporation of amino allyl-dUTP (Ambion 8439). Cy3 and Cy5 dyes (Amersham RPN 5661) were used for indirect labeling of the cDNA from reference RNA (Stratagene, Universal human reference RNA, Cat 740000) and cDNA from tumor specimens respectively. After hybridization and washing, microarrays were scanned on a GenePix 4000 microarray scanner and fluorescence ratios (tumor/reference) were calculated using GenePix software. To ensure that the measured signals reflect true readings, only spots with a ratio of signal over background of at least 1.5 in the Cy5 or 1.5 in the Cy3 channel were included. Genes were filtered retaining only those whose expression levels differed by at least 4-fold (with respect to the series average in expression level for individual genes) in at least 3 samples and those with > 70% available good data. Gene centering was applied to the expression values across this series of tumors. The filtered dataset contain a total of 1603 genes (Additional file 1). A less stringent gene filtering criteria (2-fold difference in 3 samples) was also used in an attempt to identify genes that display more subtle variations between the three BRCA1-status defined groups (6843 genes, Additional file 2). The complete gene array dataset is available through the accompanying website (http://smd.stanford.edu/).

### BRCA1 and BRCA2 status of the tumors

According to the genetic status of BRCA1 gene, we divided 28 consecutive cases of high- grade serous carcinoma of the ovary into three groups: BRCA1 loss through mutation, BRCA1 epigenetic loss and no BRCA1 loss by analyzing BRCA1 mutation, immunoexpression and promoter hypermethylation. The techniques used for evaluating the BRCA mutations, loss of heterozygosity and microsatellite instability at both loci, mRNA level of BRCA1, immunoexpression of BRCA1 and BRCA1 promoter hypermethylation of the tumors and results obtained have previously been described [16]. Briefly, BRCA1 loss through mutation is defined as germline or somatic mutation with low level of BRCA1 RNA and less than 1% nuclei positive for BRCA1 by immunohistochemistry. Cases with epigenetic BRCA1 loss show ≥4% fully methylated molecules, low level of BRCA1 RNA and less than 1% nuclei positive for BRCA1 in immunohistochemistry, with no BRCA1 or BRCA2 mutations. The no BRCA1 loss cases showed more than 1% nuclei positive for BRCA1 in immunohistochemistry and average RNA expression, and lacked either BRCA1 or BRCA2 mutations (somatic or germline) [16]. None of these tumors have BRCA2 mutations.

### Statistical analysis

Statistical analysis was performed using SPSS version 16. Fishers exact test was used to assess the association between the variables. Statistical significance was reached at p < 0.05. For gene expression profile data, unsupervised hierarchical clustering analysis and significance analysis of microarrays (SAM) were performed as described previously [20,21] and a false-discovery rate (FDR) of less than 5% was considered significant in the SAM analysis for the current study.

## Results

### Gross genomic alteration by DNA ploidy

**Figure 1:**
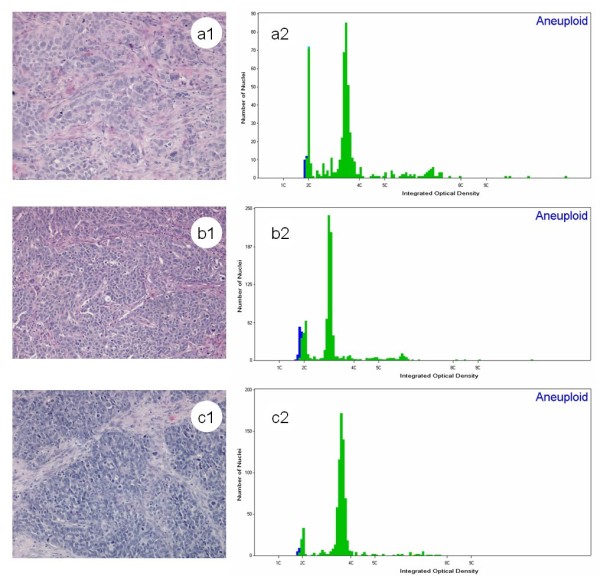
**Similar histology and histograms from high-grade serous carcinoma of the ovary with different BRCA1 status**. (a1, a2) BRCA1 loss due to genetic mutation. (b1, b2) BRCA1 loss through epigenetic promoter hypermethylation. (c1, c2) no BRCA1 loss.

DNA ploidy analysis was performed in 28 BRCA1 and BRCA 2 defined cases using an image cytometric method. The mean coefficient of variation of the diploid peaks was 3.31 (range 1.17-5.16) and that of aneuploid peaks was 3.36 (range 2.02-5.86). The mean number of nuclei analyzed was 901. Aneuploidy and tetraploidy were detected in 12 (42.9) and 7 (25%) samples respectively. The prevalence of aneuploidy and tetraploidy was not statistically different in the samples with BRCA 1 loss through mutation, BRCA 1 epigenetic loss and no BRCA loss (Table 1). The DNA index of all the aneuploid tumors was ≥ 1.4 (Figure 1) and in 2 samples it was more than 2.1. Three tumors with microsatellite instability [16] were diploid, including two with epigenetic loss (number 344 and 345) and one with BRCA1 mutation (number 223).

**Table 1:** BRCA1 status in diploid, aneuploid and tetraploid tumors.

**BRCA1 status**	**DNA ploidy diagnosis**				
	
	Diploid(%)	Aneuploid(%)	Tetraploid(%)	Total(%)	p value
BRCA1 loss through mutation	2 (25)	3 (37.5)	3 (37.5)	8 (28.6)	
BRCA1 epigenetic loss	3 (33.3)	4 (44.4)	2 (22.2)	9 (32.1)	
No BRCA1 loss	4 (36.4)	5 (45.5)	2 (18.2)	11(39.3)	0.96

### S-phase

The proliferation fraction of the tumors was evaluated manually from the histograms. S-phase fraction was divided into two groups, low and high, using median cutoff value 8.41. Even though BRCA1 loss through mutation shows low S-phase compared to others, the frequency of low and high S-phase in subgroups of BRCA1 status was not statistically different (Table 2).

**Table 2:** BRCA1 status in tumors with low and high S-phase fraction (median cut off 8.4%).

BRCA1 status	S-phase			
	
	Low (%)	High (%)	Total (%)	p value
BRCA1 loss through mutation	6 (75)	2 (25)	8 (28.6)	
BRCA1 epigenetic loss	3 (33.3)	6 (66.7)	9 (32.1)	
No BRCA1 loss	5 (45.5)	6 (54.5)	11 (39.3)	0.1

### Gene expression profile

**Figure 2:**
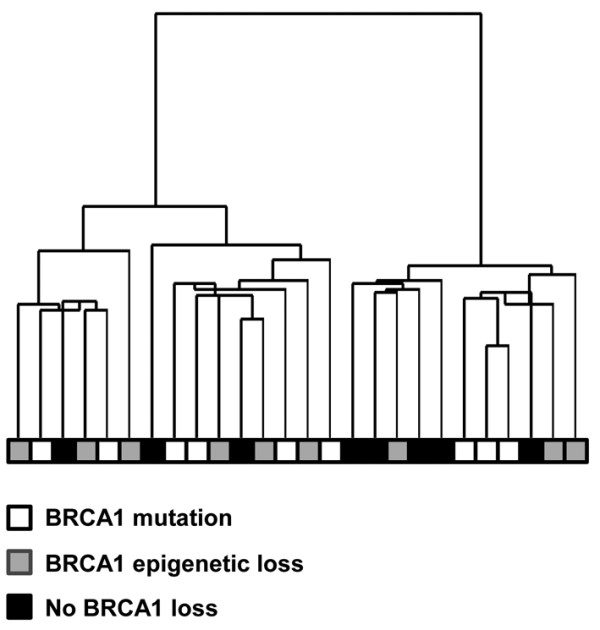
**Unsupervised hierarchical clustering of high-grade serous carcinomas of the ovary with different BRCA1 status**. Based on the expression profiles with 1603 filtered genes, there was no tendency for tumors to cluster based on BRCA1 status (BRCA1 loss through mutation, BRCA1 epigenetic loss through promoter hypermethylation, and no demonstrable BRCA1 loss). The length of the dendrogram arms is inversely proportional to the relatedness of gene expression between cases.

Global gene expression profiles could be analyzed for 26 of the 28 BRCA1 defined cases (8 with BRCA1 mutation, 9 with BRCA1 epigenetic loss through promoter hypermethylation and 9 with no demonstrable BRCA1 loss). Hierarchical clustering analysis showed no clear separation of the three BRCA1-defined groups based on the expression profiles of the filtered gene set (Figure 2). The gene expression levels of the three BRCA1-defined groups were directly compared to each other by SAM analysis. As shown in Table 3, only a small number of differentially expressed genes were identified by SAM comparison between the three BRCA1-defined groups with a FDR < 5% (list of genes with a FDR < 20% shown in Additional file 3). Comparisons between the group with BRCA1 mutation versus the group with BRCA1 epigenetic loss and between the group with BRCA1 epigenetic loss versus the group with no BRCA1 loss showed no genes with significant differential expression between the groups. Two genes (CKMT1B and KIAA1324) were found to be significantly up-regulated in the group with BRCA1 mutation compared to the group with no BRCA1 loss (FDR < 5%). No additional genes were identified to be differentially expressed (FDR < 5%) between the three groups using the less stringently filtered dataset though there was a trend for BRCA1 and a gene that is positively regulated by BRCA1, AREG [22], to be expressed more highly in the group with no demonstrable BRCA1 abnormality compared to the other groups (Additional file 4). The same trend in BRCA1 expression was also observed by qRT-PCR analysis as reported by us previously [16]. However, the expression levels of BRCA1 and AREG were not sufficiently homogeneous and distinct in each group for the difference to be identified as being statistically significant. In the case of BRCA1, this may reflect the confounding effect of the presence of normal cells in these samples, which do have BRCA1 mRNA, even though the tumor cells may lack expression.

**Table 3:** Significance analysis of microarrays (SAM) comparisons between groups with different BRCA1 status (FDR < 5%).

Comparisons	Upregulated genes	Downregulated genes
BRCA1 loss through mutation vs. BRCA1 epigenetic loss	none	none

BRCA1 loss through mutation vs. no BRCA1 loss	CKMT1B, KIAA1324	none

BRCA1 epigenetic loss vs. no BRCA1 loss	none	none

## Discussion

We found that the gross genomic alteration and gene expression profiles were similar in high- grade serous carcinoma of the ovary with BRCA1 loss through mutation, BRCA1 epigenetic loss and no evidence of BRCA1 loss. There is mounting evidence that BRCA1 plays a critical role in maintaining the genomic stability of cells [23]. Mouse embryonic fibroblasts carrying targeted deletion of BRCA1 gene were defective in a G2-M check point leading to multiple spindle poles within a single cell resulting unequal segregation of chromosomes, abnormal nuclear division and aneuploidy [5]. The mechanism of the genetic instability is caused by the failure of homologous DNA recombination, one of the pathways for the repair of double-stranded DNA breaks during DNA replication. In this process, the damaged strand is repaired using intact, homologous sequence as a template [7,8]. BRCA1 acts at the DNA damaged site as a recruiter of molecules that sense and repair DNA break and an effector of response to DNA damage during homologous recombination process [6]. In absence of BRCA1 function, the repair is through an alternate pathway, nonhomologous end joining, which is error-prone and mutagenic leading to genetic instability and aneuploidy [11].

In breast carcinoma, the total number of genomic changes, as determined by cytogenetics, was found to be almost two times higher in tumors with BRCA1 mutation than in control group [24]. In ovarian tumors, increased clonal chromosomal aberrations was observed in BRCA mutated tumors, compared to BRCA non-mutant tumors [25]. In that series, all BRCA positive tumors were serous carcinoma and the BRCA non-mutant tumors were of different histologic types. It is well known that the morphologically defined ovarian carcinomas are distinct diseases with different molecular events during oncogenesis [12], and it seems likely that this may have confounded the findings. In order to address this, we analyzed a series consisting of only high-grade serous carcinomas, excluding other subtypes (including genomically stable low-grade serous carcinomas) [19,26]. Furthermore, we have separately evaluated the tumors with BRCA1 loss due to mutation and due to epigenetic silencing due to a reported difference in prognosis [27]. In this series of 28 cases, we have observed that there is no significant difference in the distribution of aneuploidy and tetraploidy in the three subgroups. This indicates that BRCA1 inactivation is not the only mechanism for the development of aneuploidy in high-grade serous carcinoma of the ovary. Importantly, none of these cases had BRCA2 mutations that could account for chromosomal instability. In addition, all aneuploid tumors had DNA index > 1.4 indicating genomic unstable tumors [13]. Therefore, the results indicate a second currently unknown mechanism that leads to aneuploidy in ovarian serous cancer.

We observed lower S-phase in the group of tumors with BRCA1 loss through mutation compared to the other groups. The S-phase of BRCA mutant mouse embryonic fibroblast cells was significantly lower than the control cells as determined by flow cytometry[5]. However, ovarian carcinomas with BRCA germ line mutation had higher proliferation fraction than sporadic tumors as measured by Ki 67 [28].

BRCA1 and BRCA2 mutated ovarian tumors have different gene expression profiles, however, the gene expression profile of sporadic ovarian tumor overlaps with both [29]. In this study, we found essentially no differences in gene expression profile based on BRCA1 status. Only two differentially expressed genes, out of thousands examined, were identified in one of the pair-wise comparisons. This is in keeping with an earlier finding made by Tone et al on a smaller series of 13 high-grade serous carcinomas (of either ovarian or tubal origin), where highly overlapping gene expression profiles were observed between cases with known BRCA1/2 mutation and/or family history and cases with unknown familiar status [30]. These genes show no functional relationship to each other or to the genes known to be involved in BRCA function and this finding is most probably due to chance alone. While the relative small sample sizes (n = 8~9) of the different BRCA1-defined groups examined here may contributes to the paucity of consistent differences identified, it does represent the largest series examined to date and a larger number of differentially expressed genes can usually be identified between different tumor types with similar sample sizes [31,32]. Therefore, the paucity of differences observed between these groups of serous carcinomas with different BRCA1 status is likely a reflection of intra-group non-uniformity and inter-group overlap in the gene expression patterns. In addition, we recently showed that these groups of ovarian carcinoma classified based on BRCA1 status also show near-identical miRNA expression profiles [33]. This absence of distinct patterns of mRNA or miRNA expression in groups with different BRCA1 status may reflect the rapid divergence in tumors once they acquire chromosomal instability, so that every individual tumor is sufficiently unique that clustering analysis identifies no patterns. As such, their gene expression profiles irrespective of BRCA1 status all show significant dysregulation/difference from that of the putative tissues of origin in normal ovarian surface epithelium and normal fallopian tube as demonstrated previously [30,34]. This rapid divergence can also explain the dearth of differentially expressed genes on supervised (SAM) analysis, as some consistent abnormalities would have to occur within each group for there to be differences in gene expression. What this does indicate, however, is that the same abnormality, chromosomal instability, appears to be present in all groups of high-grade serous carcinoma analyzed, irrespective of BRCA1 status. While chromosomal instability can be accounted for in the BRCA1 mutant and BRCA1 epigenetically silenced groups, it will be important to identify the mechanism in the large group of tumors that lack BRCA1 or BRCA2 abnormalities and these may involve BRCA1/2-related mechanism(s) or non-BRCA1/2 related mechanism(s). PARP inhibitors have been shown to have activity in tumors with mutations of BRCA1[35]. PARP inhibitors target base excision repair mechanisms in the cell [36]. In cells that lack BRCA1 or BRCA2, homologous repair of double-stranded DNA is defective and the single strand breaks that cannot be repaired because of PARP inhibition are converted to double strand breaks in dividing cells; in the absence of BRCA proteins the double strand breaks are repaired by non-homologous mechanisms, such as non-homologous end joining, which is lethal to the cell [17,37]. Thus PARP inhibition can specifically target cells lacking BRCA, while sparing normal cells. It remains to be seen whether PARP inhibitors will be active in high-grade serous carcinomas with either BRCA1 epigenetic silencing or no evidence of BRCA1 loss, although it is conceivable that such cells may lack homologous repair functions.

## Conclusions

There was no relationship between gross genomic alteration, detected by high resolution DNA image analysis, and BRCA1 inactivation in high-grade serous carcinoma of the ovary. Gene expression profile analysis similarly revealed no significant differences between these groups. This raises two questions. What is the mechanism underlying genomic instability and development of aneuploidy in ovarian high-grade serous carcinomas that lack BRCA1 and BRCA2 abnormalities? Will these tumors, which do have genomic instability, be sensitive to therapy targeted at cells lacking in DNA repair capability, such as PARP inhibitors?

## Competing interests

The authors declare that they have no competing interests.

## Authors' contributions

MP classified the DNA ploidy histograms and drafted the manuscript. BR, CGT, CBG, MvdR helped in designing the study and drafting the manuscript. CHL performed oligonucleotide microarray experiments for gene expression profile and helped in drafting manuscript. All authors read and approved the final manuscript.

## Pre-publication history

The pre-publication history for this paper can be accessed here:

http://www.biomedcentral.com/1471-2407/10/493/prepub

## Supplementary Material

Additional file 1 **This is an excel file containing the full data for 1603 genes that met the filtering criteria**. Click here for file

Additional file 2 **This is an excel file containing the full data for 6843 genes that met a less stringent gene filtering criteria**. Click here for file

Additional file 3 **This is a table showing all the differentially expressed genes identified by significance analysis of microarray (SAM) between the different BRCA1-defined groups with a false-discovery rate (FDR) of < 20%**. Click here for file

Additional file 4 **Expression levels of BRCA1 and AREG for all cases.** Expression level is depicted compared to the mean of all samples, with green indicating lower than mean expression level and red indicating higher than mean expression level. Black indicates expression at the mean for the entire group. Gray indicates missing data. Click here for file
