# Supplementary figures and images for: Gross genomic alterations and gene expression profiles of high- grade serous carcinoma of the ovary with and without BRCA1 inactivation
Source: BMC Cancer. 2010 Sep 15;10:493. doi: 10.1186/1471-2407-10-493 (PMC2946313; doi:10.1186/1471-2407-10-493)

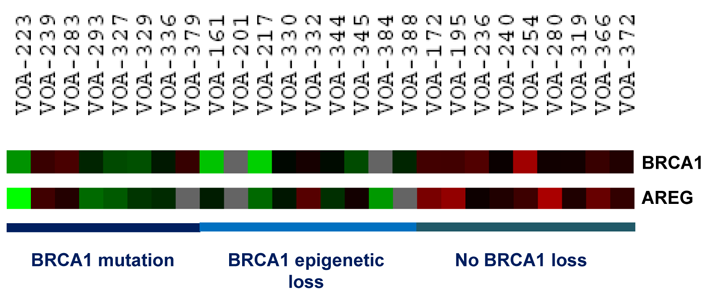

Supplement: Additional file 4 — Expression levels of BRCA1 and AREG for all cases. Expression level is depicted compared to the mean of all samples, with green indicating lower than mean expression level and red indicating higher than mean expression level. Black indicates expression at the mean for the entire group. Gray indicates missing data. [file 1471-2407-10-493-S4.TIFF]
